# Structure–Elasticity Relationships in Hybrid-Carrageenan Hydrogels Studied by Image Dynamic Light Scattering, Ultra-Small-Angle Light Scattering and Dynamic Rheometry

**DOI:** 10.3390/ma17174331

**Published:** 2024-08-31

**Authors:** Amine Ben Yahia, Adel Aschi, Bruno Faria, Loic Hilliou

**Affiliations:** 1 Laboratoire de Physique de la Matière Molle et de la Modélisation Electromagnétique, Département de Physique, Faculté des Sciences de Tunis, Campus Universitaire, Tunis 2092, Tunisia; amine.benyahia@fst.utm.tn; 2 Institute for Polymers and Composites (IPC), Campus de Azurém, University of Minho, 5800-048 Guimarães, Portugal; bruno.faria@dep.uminho.com

**Keywords:** hybrid carrageenan, image dynamic light scattering (IDLS), ultra-small-angle light scattering (USALS), pair-distance distribution function *P(r)*, rheology, strain hardening

## Abstract

Hybrid-carrageenan hydrogels are characterized using novel techniques based on high-resolution speckle imaging, namely image dynamic light scattering (IDLS) and ultra-small-angle light scattering (USALS). These techniques, used to probe the microscopic structure of the system in sol–gel phase separation and at different concentrations in the gel phase, give access to a better understanding of the network’s topology on the basis of fractals in the dense phase. Observations of the architecture and the spatial and the size distributions of gel phase and fractal dimension were performed by USALS. The pair-distance distribution function, *P(r)*, extracted from USALS patterns, is a new methodology of calculus for determining the network’s internal size with precision. All structural features are systematically compared with a linear and non-linear rheological characterization of the gels and structure–elasticity relationships are identified in the framework of fractal colloid gels in the diffusion limit.

## 1. Introduction

**Figure 1:**
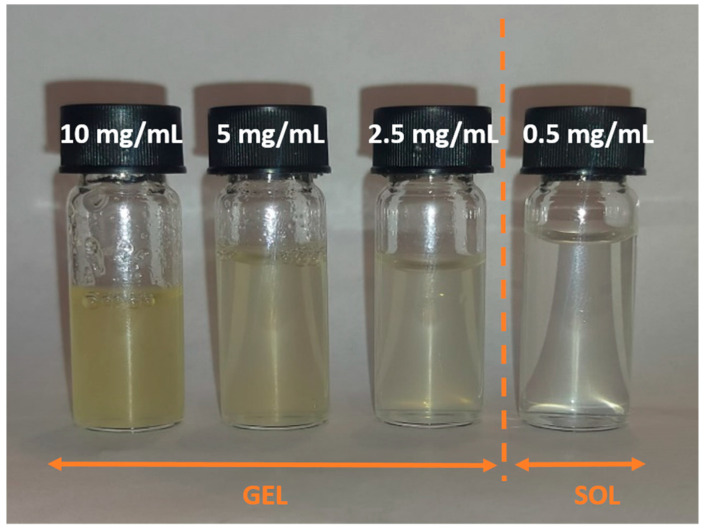
Optical appearance of KIMN samples prepared at different concentrations (indicated on covers) in phosphate buffer. All samples but the one prepared with the lowest concentration are gels.

Hydrogels are polymer networks swollen in water. The high solvent content gives hydrogels unique physical properties [1]. In spite of generally showing inferior mechanical properties when compared to chemical gels, they do combine mechanical strength and gel formation reversibility, a characteristic of particular interest when these materials are to be injected for medical of pharmaceutical applications. In general, hydrogels formed from animal or plant polysaccharides constitute an interesting class of materials frequently used in medicine and pharmacology [2,3]. Hydrogels may undergo enzymatic degradation after being injected in the body, causing the sustained and controlled release of biological-based therapeutic agents. In addition, physical hydrogels can be loaded with other materials such as DNA, of interest for tissue engineering and regenerative medicine [2,3,4,5,6]. The hydrogel microstructures most often used in life sciences are three-dimensional matrices for specific applications such as tissue engineering or intelligent drug delivery [4,5,6]. Hydrogel properties are linked to its internal dynamics. While bulk hydrogel dynamics have been studied extensively, how hydrogel networks respond locally to deformation has yet to be understood. This is particularly the case for hybrid-carrageenan gels, a special class of the kappa-carrageenan family, for which structure–elasticity relationships are not firmly established [7]. Hybrid carrageenans are copolymers made of the statistical distribution of blocks of kappa-carrageenan, blocks of iota-carrageenan and in special cases non-gelling disaccharide units of the kappa-carrageenan family such as nu-carrageenan or mu-carrageenan. The full chemical structure of this class of polysaccharide has been reviewed recently by Souza et al. [7], where Figures 1 and 3 give full detail about the differences in the chemical structures of the kappa-carrageenan family.

In this work, a hybrid carrageenan (KIMN) extracted from the seaweed *Mastocarpus stellatus* [8] was used to produce hydrogels. This specific polysaccharide presents advantages when compared to the kappa- and iota-carrageenans extensively used in industry and studied in the scientific literature. Hybrid carrageenan forms more elastic gels than those made with iota-carrageenan but does not show water syneresis [7]. Such water release often shows up during the formation of kappa-carrageenan gels upon cooling of hot solutions in the presence of salt and is the source of experimental artefacts when hydrogels are sheared to measure elastic properties [9]. To study the microstructural and macroscopic properties of hybrid carrageenan gels, two major speckle imaging techniques were used: image dynamic light scattering (IDLS) and ultra-small-angle laser light scattering (USALS). The implementation of USALS and IDLS techniques for the study of the complexation of coacervates is described in a recently published article [10]. Here these techniques are used to explore the multilength structures of KIMN hydrogels prepared with different concentrations, ranging from 0.5 to 10 mg/mL. Indeed, using USALS and IDLS can effectively evaluate local hydrogel structures [11,12,13,14,15] which then can be opposed to the mechanical properties of gels studied by dynamic rheometry in the frame of theories for fractal colloidal gels [16,17,18]. To the best of our knowledge, this is the first work where these cited techniques were employed to investigate the structure of gels formed by a hybrid carrageenan in view to elucidate the structure–elasticity relationships.

Indeed, there are too few reports where both elasticity and structure of carrageenan gels are simultaneously studied to identify structure–elasticity relationships. The fibrillary structure of carrageenan gels has long been established by techniques such as electronic microscopy [18,19] or more recently atomic force microscopy [20] as well as scattering techniques [21]. Essentially, kappa-carrageenan forms large fibrillary aggregates which are thought to be responsible for the strong elasticity and brittleness of the network. In contrast, iota-carrageenan self-assembles in finer fiber-like aggregates which confers a weaker elasticity to a loose network showing more strain resistance [20,21,22]. However, none of the published studies rationalized the gel structure and mechanical properties by molecular or fiber network theories, see [22] and references therein. For hybrid carrageenan, the network gel structure is overall fiber-like with strands connected in a three-dimensional network, but the mechanism for self-assembly is unknown [7], and structure–elasticity relationships were only explained by theories designed for fractal colloidal gels [18].

## 2. Materials and Methods

To extract carrageenan, a protocol described at length elsewhere was employed [8]. In brief, 2 g of dried seaweed was soaked in 50 mL of Milli-Q water at 80 °C for 3 h. Then, 50 mL of distilled water was added to the suspension and the mixture was heated to 90 °C for 1 h for carrageenan extraction. The viscous suspension was centrifuged at 8000 rpm for 10 min and oven-dried at 50 °C overnight.

The resulting carrageenan is a statistical block copolymer made of 59 mol.% kappa-carrageenan blocks, 22 mol.% iota-carrageenan blocks, 12 mol.% mu-carrageenan disaccharides and 6 mol.% nu-carrageenan disaccharides, as shown by the proton NMR spectrum measured at 70 °C in a 1 wt.% solution in D_2_O using a Bruker Avance III (Billerica, MA, USA) at 400 Mhz, and displayed in Figure S1.

The stock solutions of KIMN were prepared with hot phosphate buffer and subsequently diluted to obtain concentrations between 0.5 and 10 mg/mL. Figure 1 shows that the hydrogel formed after cooling of the hot solution is opaque for *c* > 0.5 mg/mL. This indicates that multiple scattering is not negligible and the IDLS and USALS experiments are suitable for studying the structure of the formed hydrogel.

To study the dynamics of gels where their Brownian motions are very slow, we used the technique of image dynamic light scattering (IDLS). In this technique we used a laser of wavelength *λ* equal to 632.8 nm with a continuous wave output power of 50 mW. The beam coming out of the laser is focused on the sample holder cell. A diaphragm with an aperture diameter of 0.8 mm is used to limit the region measured during the experiments. The light scattered by the studied samples is collected by a lens with a focal length of 110 mm and is captured by a high-speed monochrome CCD camera (Pulnix TM 6470 America, Sunnyvale, CA, USA, total effective area of 640 × 480 pixels) fixed at an angle of 45° with respect to the incident light. The scattering volume of the studied sample is located at a distance *D* from the object plane of the camera. The lens fixed on the CCD camera is used to reduce the effect of stray light. In the conventional DLS method, the detector must have the same size as the coherence area (maximum signal-to-noise ratio). In this method, the mesh size must define the same coherence area and the area reduction is ensured by the small pixel size. The smallest area for a pixel in the CCD camera is 7.4 μm × 7.4 μm. For our experimental setup, the distance *D* between the detector and the scattering volume is equal to 180 mm and *d* is the diameter of the scattering volume, *d* ~1 mm. The coherence area *A_ch_*, also called speckle size, can be determined by the relation *A_ch_ =* (4*λ*^2^*/π*)(*D/d*)^2^. In our case, the speckle size is approximately equal to 16,519 μm^2^, i.e., the coherence area occupies 128 × 128 μm^2^ (~17 × 17 pixels) in the CCD camera sensor.

Ultra-small-angle light scattering (USALS) is more suitable for studying microscopic-scale structures such as concentrated polymers, turbid solutions of colloids and gels. This device is equipped with a 20 mW helium–neon laser with a wavelength of 632.8 nm (Cube Coherent Inc.). The same camera collected the same sequences of images as that used for IDLS. This apparatus operated at small angles and covered a range of scattering vector *q* = (4*πn/λ*)*sin*(*θ*/2), where *λ* represents the wavelength, *n* is the refractive index and *θ* refers to the scattering angle, from 0.1 to 1.6 μm^−1^. All obtained USALS images were analyzed using ImageJ with standard protocols to subtract the background and remove the beam stop from the raw images.

The rheological characterization of gels was carried out with a stress-controlled rotational rheometer (Anton Paar MCR302, Graz, Austria). The temperature control system is a Peltier with an electrically heated hood (P-ETD300 and A-ETD300, Anton Paar, Graz, Austria). Hot solutions prepared from the stock solution were loaded at 85 °C under the plate geometry (diameter 40 mm) and dodecane was used to cover the rim of the sample and avoid water loss. All tests were performed between 250 and 300 microns with a gap controlled by the normal force set to 0 N during the cooling of the hot solution from 85 °C to 30 °C in 1 h. This gap control allows the reliable determination of the linear viscoelastic properties of gels [9]. Right after cooling, the mechanical spectrum of the formed gel was recorded by sweeping the frequency from 100 Hz to 0.01 Hz with a strain of 0.1%. Finally, a sweep in the amplitude of the oscillatory shear strain was performed from 0.01% to 1000% (20 data points per decade) with a frequency of 1 Hz to confirm that measurements during the recording of the mechanical spectrum at 25 °C were carried out within the linear regime of viscoelasticity and additionally to characterize the large deformation behavior of the gels.

## 3. Results and Discussion

### 3.1. Image Dynamic Light Scattering (IDLS)

The IDLS technique is generally used for a non-ergodic medium where the system exhibits non-exponential relaxation as usually observed near glass or sol–gel transitions. This experimental technique is described in detail elsewhere [10]. The stretched exponential function proposed by Kohlrausch, Williams and Watts (KWW) commonly describes this non-exponential relaxation. The intensity correlation function, *C*(*τ*), is given by the following equations [10]:

(1) $$C\left(\tau \right)=A+B{\left[C_{0}(\tau )\right]}^{2},$$

(2) $$C_{0}(\tau )=exp{\left[-\frac{\tau }{\tau_{0}}\right]}^{\alpha }$$

where *A* and *B* are constants, *α* and *τ*_0_ are the stretching exponent and the characteristic relaxation time, respectively, and *τ* is the time [16]. Note that the stretching exponent *α* is generally linked to the width of the relaxation time distribution. For example, *α* = 1 corresponds to a single relaxation time, whereas the width of the distribution increases as *α* decreases below 1. In contrast to this, *α* = 2 corresponds to a Gaussian distribution. Functional forms with *α* between 1 and 2 are called compressed exponentials, rarely observed in colloidal systems and they are related to supradiffuse dynamics [17,23,24]. Values of *α* ≤ 1 are commonly observed in polymer-based systems that are close to glass transition [14,25].

**Figure 2:**
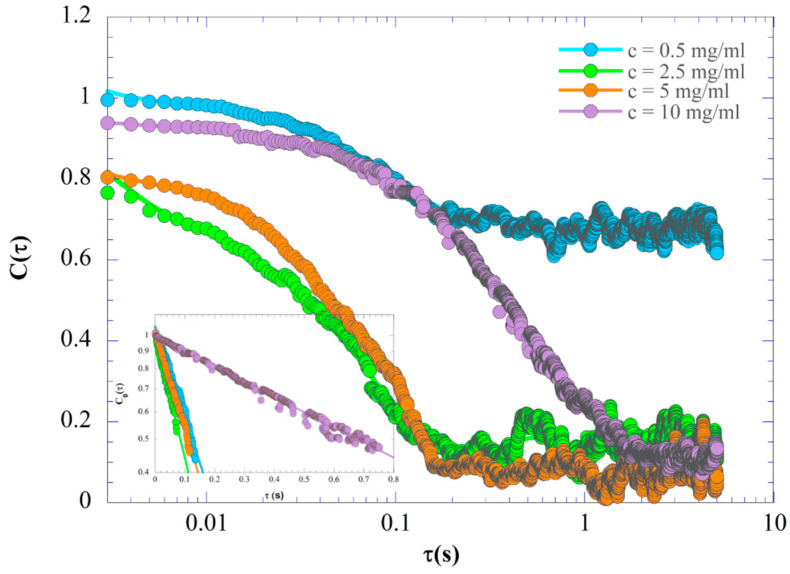
The time correlation function of the scattering intensity, *C*(*τ*), of the KIMN samples prepared with four different concentrations and measured at a temperature of 20 °C. The inset presents the semi-logarithmic variation of *C*_0_(*τ*) vs. *τ* (see Equation (2)).

Figure 2 shows the intensity correlation function *C*(*τ*) change versus time for samples prepared with different KIMN concentrations. It is clear from the figure that the relaxation time becomes shorter for the concentration range between 0.5 and 2.5 mg/mL (sol–gel transition) and then longer for higher concentrations *c*, i.e., *c* > 2.5 mg/mL. The image correlation function (ICF) exhibits power-law behavior given by Equation (1), see the inset in Figure 2. At the same time, the intercept of the ICF at *τ* = 0 begins to deviate from unity. In other words, the initial amplitude of the ICFs was decreased significantly from unity at the high concentration (*c* ≥ 2.5 mg/mL). This is an indication of the appearance of non-ergodicity. The same result was obtained by Shibayama et al. by time-resolved dynamic light scattering (TRDLS) measurement on a polymer gel [26]. Table 1 summarizes the results obtained from fitting the IDLS data to Equation (1). In Table 1, the increase in relaxation time *τ*_0_ is observed for very high concentrations (*c* > 5 mg/mL). Note that this increase extends over an order of magnitude, indicating that the size of the objects that scatter light to form the imaged speckles become enormous compared to the size of the individual carrageenan chains (~180 nm) [27] or carrageenan strands in the gel structure (100 nm [22]).

The exponent *α* increases as the concentration increases and shows an unusual value greater than 1 for the concentration *c* = 10 mg/mL. During a study on gels formed by colloidal aggregation, authors found a value close to the exponent *α* ≈ 1.35 [28], while others obtained a somewhat larger value, *α* = 1.5 [29,30] and they concluded that these values correspond to a colloidal fractal gel. The results in Table 1 indicate that the whole hydrogel structure of KIMN is frozen and, due to its non-ergodic nature, shows self-similarity from local to macroscopic scales. In addition, these results suggest that the neighboring scattering objects partially interpenetrate each other in the highly concentrated states. Kureha et al. [31], in a DLS study on a microgel suspension, explained this phenomenon by the fact that microgel particles may be trapped in a cage formed by neighboring microgels and cannot move over long time scales. Carrageenan gels are seen more as a network of fiber-like objects [7,22] and due to the length and time scales probed by the IDLS technique, we cannot have the details of the fibrous structure, but rather a structure on a larger scale, i.e., on the scale of clusters of fiber-like objects. We can use fractal colloidal gel concepts for the assembly of such round clusters as proposed in an earlier work [18] where the non-linear rheology of KIMN gels formed in KCl was correlated with the fractal dimension of the structure.

### 3.2. Ultra-Small-Angle Light Scattering (USALS)

**Figure 3:**
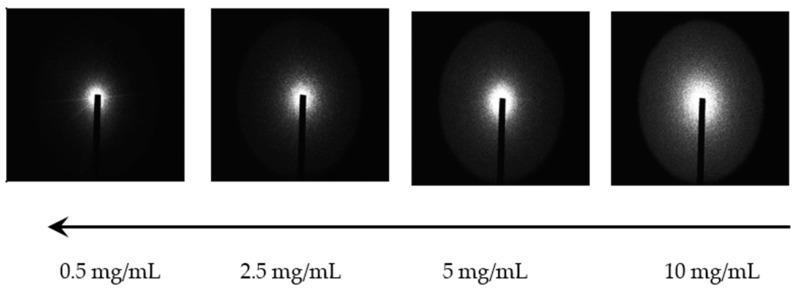
Comparison of the two-dimensional isotropic light scattering (USALS) patterns obtained for different KIMN concentrations at 20 °C.

Using USALS, the evolution of the structural organization of KIMN solutions and gels through the sol–gel transition can be studied by acquiring scattered images at different times at a fixed point of the optical cell. USALS, along with small-angle X-ray or neutron scattering, allows one to obtain more information about the structure of fractal objects as it explores the reciprocal space *q*. Fractal dimensions can be calculated from slopes of log–log plots of scattered intensity versus scale. As irregular, complex shapes, fractals possess two important properties, notably self-similarity and scale invariance, and can be characterized by their degree of irregularity or complexity using fractional dimensionality (also known as their fractal dimensions introduced by Mandelbrot) [32]. The USALS technique has been carried out in our laboratory using a setup developed by Ferri [33]. The experimental technique and the theory are described in detail elsewhere [10]. Figure 3 shows the resulting light scattering patterns as a function of hybrid-carrageenan concentration.

**Figure 4:**
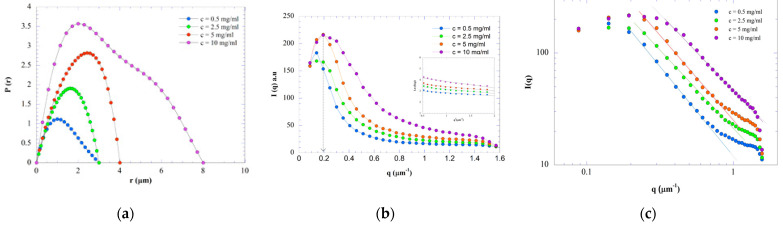
Analysis of the USALS patterns displayed in Figure 3: (**a**) Pair-distance distribution functions *P(r)* computed from the intensity profiles *I*(*q*) of samples with varying concentrations in hybrid carrageenan; (**b**) Intensity profiles with inset presenting a Guinier plot; the vertical arrow indicates the location of the Bragg correlation length *Λ*, see text; (**c**) Intensity profiles plotted in log–log scales to enable the determination of the fractal dimensions *D_f_* (lines are fits of power laws to the data, see text).

The patterns are isotropic, suggesting that the light is scattered by isotropic objects (spherical clusters) and the light is more scattered by more concentrated samples, which also results in an increase in the intensity *I*(*q*) for all *q*, see Figure 4.

This increase may result from the concentration-dependent increase in the number of scatterers. Some researchers studying the phenomenon of phase transitions in self-assembling biopolymers [34,35,36] have reported similar behavior of diffusion patterns and explained it as a signature of multichain association.

ImageJ (concentric circles plugins) software (version 1.53k) was used to extract USALS intensity profiles from the scattering patterns measured at different concentrations (T = 20 °C) and the possible conformation of hybrid carrageenan can be estimated as a first approximation from the shape of the pair-distance distribution function *P(r)*, calculated from the form factor *P(q)* by indirect Fourier transform (IFT) analysis. Figure 4a shows the unnormalized distribution functions at different values of r. *P(r)* is sensitive to concentration effects and sample polydispersity. Not all profiles are symmetrical, manifesting the non-spherical distribution of colloidal density. In addition, the difference in the size is clearly visible as the profiles reach zero values at different values of *r* labeled here as *D_max_*, *P(D_max_) = 0*. Figure 4a shows that *D_max_* abruptly grows when the concentration increases, see the corresponding values reported in Table 2. *D_max_* gives an idea of the maximum diameter of the scattering round clusters. In addition, for the first two concentrations (0.5 and 2.5 mg/mL) the *P(r)* curve has a fairly broad, bell-shaped maximum. The maximum in *P(r)* is also shifted with the concentration towards distances less than *D_max_*/2 and this corresponds to a network composed of flattened particles [37].

For 5 mg/mL, the maximum of the distribution seems to have a characteristic size much greater than *D_max_*/2. A maximum displacement towards distances greater than *D_max_*/2 generally indicates a network composed of carrying troughs. For 10 mg/mL, the *P(r)* curve presents two maxima. These two maxima may indicate the presence of two heterogeneous, rather elongated objects differing in dimensions. Alternatively, the second peak can be seen as a shoulder and could be indicative of a more flexible fiber network as shown in a study on elongated colloids [38]. A similar profile for *P(r)* has been found in a study on biomaterials made of polysaccharides and oligopeptides, which was attributed to the existence of a fibrous network [39].

Figure 4b shows *I*(*q*), the variation in the wave vector *q* of the scattering intensity of the four KIMN samples measured at T = 20 °C. As the concentration increases, the *I*(*q*) plots exhibit extreme scattering at low *q*. This indicates a variation in the correlation length of the formed gel network. There are different types of non-homogeneities in hydrogels, such as spatial, topological and connectivity non-homogeneities [40]. These non-homogeneities can be characterized by scattering measurements such as the DLS technique. USALS is one of the best techniques for studying gel non-homogeneities because it provides information on spatial concentration fluctuations and concentration differences between solvent-rich and solvent-poor regions. Based on the results of the pair-distance distribution functions, the USALS profile of the KIMN samples could be successfully fitted using the expression of Guinier approximation, in BioXTAS RAW software (version 2.3.0) [41], at low *q* range (*qR_g_* < 1, with *R_g_* the radius of gyration of the fractal clusters scattering the light):

(3) $$I(q)=I_{Gui}(0)e^{\frac{-q^{2}{R_{g}}^{2}}{3}},$$

where *I_Gui_*(0) is the intensity at *q* = 0. The inset to Figure 4b illustrates the fitting and the computed values for *R_g_* are reported in Table 2. The Guinier terms (*R_g_* and *I_Gui_*(0)) increase with the concentration, indicating the increase in the average size and quantity of aggregates formed. This can be explained by the large aggregation of polysaccharide particles making up the gel network. At the lowest KIMN concentration (*c* = 0.5 mg/mL), the Debye–Bueche (D-B) equation [42] was more adapted for the modeling of the scattering profile. This equation can describe the *q* dependence of the light scattered by a medium with non-homogeneities with correlation length *ξ_DB_* and reads:

(4) $$I(q)=\frac{I_{DB}(0)}{{\left(1+q^{2}{\xi_{DB}}^{2}\right)}^{2}},$$

with *I_DB_*(0) the intensity of the light scattered at *q* = 0. The value of *ξ_DB_* computed from the fitting of Equation (4) to the intensity profile at *c* = 0.5 mg/mL is also reported in Table 2. Rivas-Araiza et al. explained *ξ_DB_* for an isotropic hydrogel formed by chitosan and for low concentrations as the correlation length between clusters of particles [13]. In addition, at higher KIMN concentrations (*c* > 0.5 mg/mL) a peak in the intensity shows up at *q_max_* and indicates a Bragg correlation length denoted *Λ* and given by *Λ =* 2*π/q_max_*. *Λ* here should represent the correlation length for regions of a distinct two-phase structure [43]. The distinct two-phase regions may emerge at a later stage of gelation. Furthermore, the characteristic size decreases as the concentration increases (Table 2). The obtained results show a transition of microstructural morphology from a solution formed by random coil chains (*c* = 0.5 mg/mL) [26] to gels with a fibril network (*c* > 0.5 mg/mL) [8]. With the presence of the phosphate buffer, a non-homogeneous self-assembly reaction occurs, thus creating heterogeneities in the carrageenan gels. In the gel state, concentration fluctuations cannot be released and remain in the form of frozen heterogeneities (i.e., permanent concentration fluctuations). Mitsuhiro Shibayama explained the change of correlation length, during a study on polymer gels, by the fact that when the polymer swells, the first chemical neighbor bonding groups become “visible” by scattering. The small clusters penetrating the large clusters in the reaction bath are separated into phases by swelling of the interstitial medium [44].

To determine the small-scale (thus large *q* regime) structural changes with the increase in KIMN concentration in the gels, the experimental data were approximated by the power-law dependence of the intensity values with *q,* that is, *I*(*q*) *~ q*^−*D*^*_f_*. According to Porod’s law, the value of the power-law exponent *D_f_* is linked to the volumetric fractal dimension (mass or porous) of the dispersed clusters of particles [45,46]. From the slope of the linear curves of Figure 4c which presents log–log plots of the scattered intensity, we were able to determine the fractal dimension *D_f_* of the KIMN gels at different concentrations. The slope of the linear region decreases, indicating a decrease in the fractal dimension *D_f_* as the concentration increases. Based on the Porod approach related to the fractal dimension, it was found that with an increase in concentration, the level of swelling of the polymer coil increases, as indicated by the decrease in the exponent *D_f_* of the value of 1.7 (excluded volume effect forms) to 1.5 (DLCA). In fact, in the case of *c* > 0.5 mg/mL, the gel is loose, and the aggregation kinetics is diffusion-limited (DLCA). Even for high concentrations, the hybrid carrageenan is not consequently more compact and the gel kinetics is not reaction-limited (RLCA). Authors have explained that this decrease in *D_f_* is due to the increase in the phase separation of KIMN from the solvent and this leads to coarsening of the internal structure of the cluster [47]. Other authors have explained the low value of the fractal dimension by the increase in the characteristic size in clusters where the excluded volume interactions are very weak and by the loss of conformational entropy [48,49,50,51].

### 3.3. Rheological Properties of Gels

**Figure 5:**
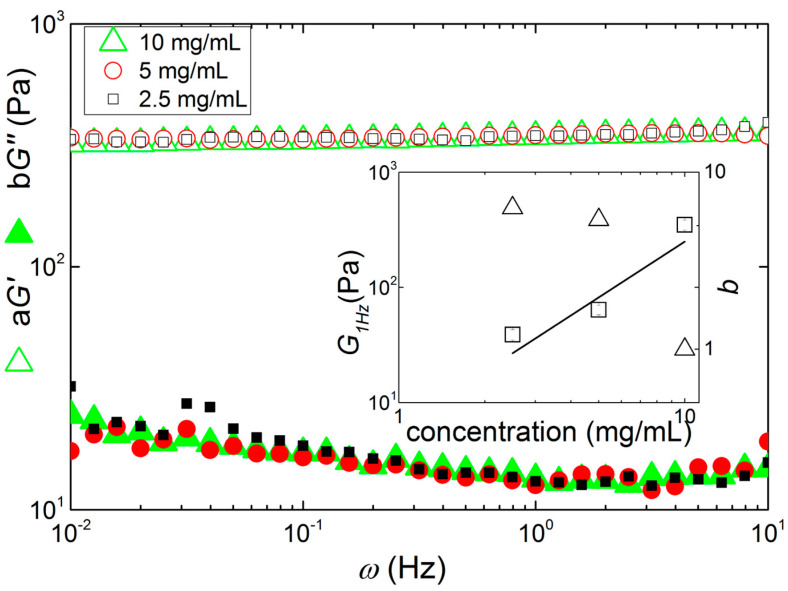
Master curve of mechanical spectra of KIMN gels measured at 30 °C by cooling hot solutions with different polysaccharide concentrations: 2.5 mg/mL (squares), 5 mg/mL (circles) and 10 mg/mL (triangles). Superimposition of curves is achieved by vertically shifting moduli *G*′ and *G*″ by a factor *a* or *b*, respectively. Inset: concentration dependence of the shift factor *b* (triangles) and of modulus *G*′ measured at 1 Hz (*G*_1*Hz*_, squares), which also represents the concentration dependence of the shift factor *a* since *G*″ is virtually non-frequency-dependent. The line indicates a power law fit to the *G*_1*Hz*_ data with computed exponent 1.61 ± 0.75.

The mechanical spectra of the gels measured at 30 °C are presented in Figure 5. Overall, all gels show similar spectra as they all can be superimposed on a master curve by vertically shifting *G′* and *G*″ measured at lower concentrations onto the spectrum measured for 10 mg/mL. This indicates that the gels formed at all tested concentrations show identical mechanically relevant structures from the nanoscale to the microscale, which are the length scales typically probed by dynamic rheometry. Note that no gel could be measured at a concentration of 5 mg/mL, which is in harmony with the coil-like conformation of the polymer chains found in USALS and IDLS.

The inset to Figure 5 reports the elasticity of the gels, estimated from the value of *G*′ measured at 1 Hz, as a function of the concentration in polysaccharide. A power law fit to the data returns an exponent of 1.61 ± 0.75, which is in harmony with many of the exponents found in the literature for carrageenan gels at equivalent salt and polymer concentrations [22]. Interestingly, this exponent gives a fractal dimension *D_f_* in the range 1.2 to 1.5 for strands making up a frozen network [52] or for colloidal gels with similar fractal dimension for aggregating clusters and structural units making up the cluster [53]. This theoretical interpretation of the rheological data displayed in Figure 5 is in agreement with both USALS and IDLS data as a similar fractal dimension for the network was found and a similar non-dependence on the concentration was inferred, see Table 2.

**Figure 6:**
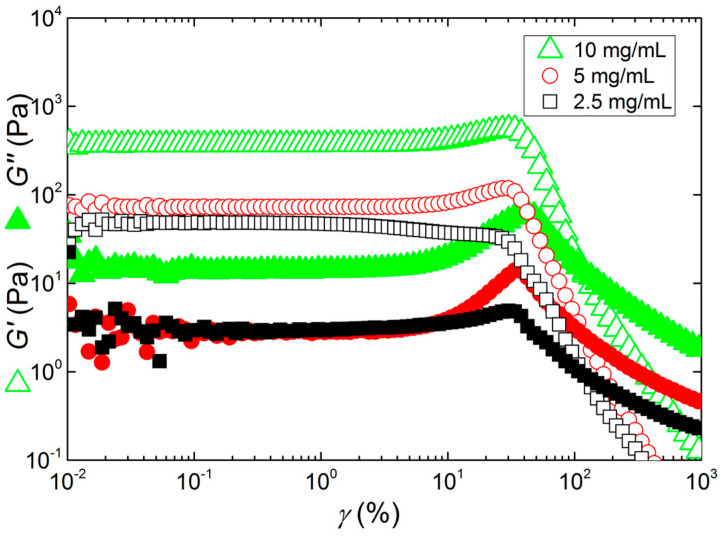
Responses of the KIMN gels prepared at different KIMN concentrations to a sweep in the amplitude of the oscillatory shear strain *γ* applied with a frequency of 1 Hz.

The large deformation behavior of the gels is also in favor of the picture of a network made up of aggregating clusters built by rigid rod-like strands. For such architecture, and under the assumption of strong links between mechanically weaker clusters, a strain hardening behavior is expected [54]. Figure 6 indeed illustrates such strain hardening for the two gels formed at higher concentrations. The strain hardening is mirrored in the increase in *G*′ as the shear strain is ramped to values larger than the strain limit of the linear viscoelastic regime. After the strain hardening, a local maximum in *G*′ shows up before the latter starts to fall at larger strains. In the meantime, *G*″ passes through a maximum suggesting that the drop in *G′* coincides with a non-recoverable or irreversible strain-induced process. A recent rheological study with KIMN gels in KCl and NaCl showed that after the maximum in *G*″, the reversibility in the large-amplitude oscillatory shear (LAOS) behavior was lost [55]. Indeed, applying a LAOS sweep right after a sweep performed up to strain amplitudes larger than the amplitude where *G*″ peaks results in the measurement of a gel with significantly reduced elasticity and showing no more strain hardening.

## 4. Conclusions

This work studied the effect of concentration on the microstructural morphology and rheology of hybrid-carrageenan physical gels. The properties of the sol–gel phase transition process, carried out using the IDLS and USALS techniques, allow an exhaustive study of the behavior of the gels within the theoretical framework of colloidal gels. Within this theoretical framework, the structures of hybrid-carrageenan gels probed here for the first time by IDLS and USALS nicely correlate with their rheological characteristics. These results thus bring novelty for the specific case of hybrid carrageenans. The relaxation time, the radius of gyration, the fractal dimensions, correlation length and internal structure of KIMN clusters were determined from IDLS and USALS data.

The main conclusions from this study are listed here, from the most important to least important results.

– Based on the Porod approach related to the fractal dimension of the network, *D_f_*, it was found that with an increase in concentration, the level of swelling of the polymer clusters increases, leading to the decrease in the exponent *D_f_* from the value of 1.7 (excluded volume effect forms) down to 1.4 (diffusion limited aggregation). These values coincide with the *D_f_* computed from the rheological data.
– In more concentrated solutions, fewer hybrid-carrageenan particles are present, i.e., they are made of dense phase, contrary to the more dilute solution. The correlation size determined by the Debye–Bueche approximation for the dilute solution and by *Λ* for the gels shows a microstructural transition from isotropic suspension of clusters to a network of fibrils. Quantifying the size of hybrid-carrageenan hybrid clusters using the USALS technique encompasses the consideration of the Guinier approximation to determine their radius of gyration *R_g_*. The values obtained for *R_g_* are an indicator of the size of the clusters and showed that with an increase in the concentration of the hybrid carrageenan, this size increases.
– Overall, the results revealed that USALS measurements can provide substantial structural information on hybrid-carrageenan hydrogel systems which explains the strain hardening of the stranded fractal network when submitted to large-amplitude oscillatory shear.

In the case of the more diluted sample, the diffusion of light scatterers reminiscent from the classical case of colloidal particles was observed by IDLS. The cooperative diffusion of polymers within particles results from thermal fluctuations of the polysaccharide chains. In the high-concentration region, the hydrogel was considered a macrogel because non-ergodicity appeared and the slow modes did not decay.

From the point of view of the design of hydrogel matrices in biomedical applications, biological experiments will be necessary to evaluate the microstructural morphology and resulting linear and non-linear elasticity of these materials. The information obtained on the topology of these hydrogels is relevant for predicting the cross-linked structure and controlling, for example, the drug release kinetics. This type of hydrogel could be applied in the field of nerve regeneration, in combination with neural cells or Schwann cells.

## Figures and Tables

**Table 1:** Results obtained from the fit of Equation (1) to the IDLS data plotted in Figure 2.

*c* (mg/mL)	*τ*_0_(s)	*α*
0.5	0.13	0.69
2.5	0.11	0.93
5	0.17	1.06
10	1.32	1.37

**Table 2:** Structural parameters extracted from the USALS experiments on hybrid-carrageenan samples at different concentrations *c* in phosphate buffer: distance *D_max_* where the pair-distance distribution function *P(r)* falls to 0, *R_g_* and *I_Gui_*(0) are respectively the radius of gyration of the fractal clusters and the light intensity scattered at *q* = 0 computed from a fit of Equation (3) to the data, *ξ_DB_* is the correlation length between non-homogeneities computed from a fit of Equation (4) to the data, *Λ* is the Bragg correlation length signaled by the wave vector where the light intensity passes through and maximum and *D_f_* is the fractal dimension of clusters of particles computed from a power law fit to the data at lower *q*.

*c* (mg/mL)	*D_max_* (μm)	*R_g_* (μm)	*I_Gui_*(0) (a.u.)	*ξ_DB_* (μm)	*Λ* (μm)	*D_f_*
0.5	3	0.89	23.01	3.6	n.a. *	1.7
2.5	3	1.19	47.67	n.a. *	39.3	1.5
5	4	1.95	57.5	n.a. *	32.6	1.4
10	8	2.74	244.67	n.a. *	32.6	1.4

* Parameter not computed.

## Data Availability

The raw data supporting the conclusions of this article will be made available by the authors on request.

## Supplementary Materials

The following supporting information can be downloaded at: https://www.mdpi.com/article/10.3390/ma17174331/s1, Figure S1: Proton NMR spectrum of the hybrid carrageenan extracted from Mastocarpus stellatus at 70 °C with a 1 wt.% solution sample in D2O (with corresponding peak used as a reference [56]). Highlighted peaks with respective integrated signals are assigned to ν-carrageenan (5.55 ppm), ι-carrageenan (5.33 to 5.3 ppm), μ-carrageenan (5.28 to 5.24 ppm) and κ-carrageenan (5.1 ppm).
