# Supplementary material for: Structure–Elasticity Relationships in Hybrid-Carrageenan Hydrogels Studied by Image Dynamic Light Scattering, Ultra-Small-Angle Light Scattering and Dynamic Rheometry
Source: Materials (Basel). 2024 Aug 31;17(17):4331. doi: 10.3390/ma17174331 (PMC11395807; doi:10.3390/ma17174331)
Supplement: Supplementary file 1 [file materials-17-04331-s001.zip › materials-3155768-supplementary.pdf]

SUPPLEMENTARY MATERIAL

Structure-elasticity relationships in hybrid-carrageenan hydrogels studied by Image Dynamic Light Scattering, Ultra-Small Angle Light Scattering and dynamic rheometry

Amine Ben Yahia [1], Adel Aschi [1,*], Bruno Faria [2] and Loic Hilliou [2,*]

[1] Laboratoire de Physique de la Matière Molle et de la Modélisation Electromagnétique, Département de Physique, Faculté des Sciences de Tunis, Campus Universitaire, Tunis 2092, Tunisia; amine.benyahia@fst.utm.tn

[2] Institute for Polymers and Composites (IPC), Campus de Azurém, University of Minho, 5800-048 Guimarães, Portugal; bruno.faria@dep.uminho.com

* Correspondence: aschi13@yahoo.fr (A.A.); loic@dep.uminho.pt (L.H.)

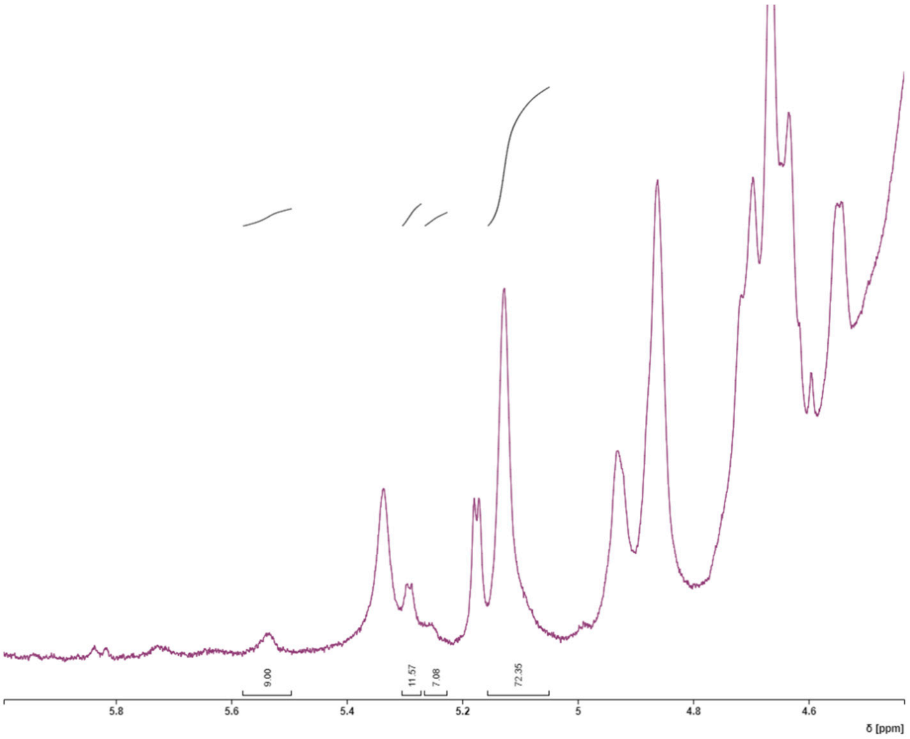

Figure S1. Proton NMR spectrum of the hybrid carrageenan extracted from *Mastocarpus stellatus* at 70 ºC with a 1 wt.% solution sample in D_2O (with corresponding peak used as a reference [S1]). Highlighted peaks with respective integrated signals are assigned to ν-carrageenan (5.55 ppm), ι-carrageenan (5.33 to 5.3 ppm), μ-carrageenan (5.28 to 5.24 ppm) and κ-carrageenan (5.1 ppm).

References

[S1] van de Velde, F., Pereira, L., & Rollema, H. S. The revised NMR chemical shift data of carrageenans. *Carbohydrate Research* **2004**, *339*, 2309-2313.
